# Complete Genome Sequence of a Putative New Bacterial Strain, I507, Isolated from the Indian Ocean

**DOI:** 10.1128/genomeA.00246-18

**Published:** 2018-04-19

**Authors:** Shu-yan Wang, Jia-qiang Wei, Hao Chen

**Affiliations:** a Key Lab of Marine Bioactive Substances of SOA, The First Institute of Oceanography, Qingdao, China; b Laboratory for Marine Biology and Biotechnology, Qingdao National Laboratory for Marine Science and Technology, Qingdao, China; c Qingdao Key Lab of Marine Natural Products R&D, Qingdao, China

## Abstract

Bacterial strain I507 was isolated from the central Indian Ocean and may be a potential novel species, according to the 16S rRNA gene sequence. Here, we present its complete genome sequence and expect that it will provide researchers with valuable information to further understand its classification and function in the future.

## GENOME ANNOUNCEMENT

A putative new bacterial strain, I507, was isolated from 500 m below sea level in the central Indian Ocean (21.70°S, 78.85°E). Based on the results of 16S rRNA gene sequence analysis, this strain had the closest phylogenetic relationship with the genus Devosia (98%), but the highest similarity was observed with several uncultured bacterium clones (GenBank accession numbers KJ549089, KM382235, and HQ697727) (1). Here, we acquired the isolate after cultivation in 2216E medium (5% [wt/vol] peptone, 1% [wt/vol] yeast extract, and 20% [wt/vol] NaCl) at 28°C for 3 days and sequenced its complete genome using PacBio technology.

Genomic DNA was extracted using a cetyltrimethylammonium bromide (CTAB)-based extraction buffer (2). Concentration and quality of the samples were determined using a Qubit 3.0 fluorometer (Life Technology) and 0.6% agarose gel, respectively. Genome sequencing was performed at Frasergen Information Co. Ltd. (Wuhan, China) using PacBio long-read sequencing technology. A PacBio standard genomic library was sequenced on single-molecule real-time (SMRT) Cell 1M version 2.0. *De novo* genome assembly was performed using the Hierarchical Genome Assembly Process (HGAP) version 3.0 (3). The assembled genome was submitted to the NCBI Prokaryotic Genome Annotation Pipeline for gene annotation (4).

Finally, there were 72,791 subreads produced by the PacBio platform, which were assembled into one contig using the HGAP software. The total length of the complete genome was 4,005,916 bp, with a G+C content of 61.93%. The genome coverage was 177.0×. The genome contained 3,917 genes and 3,859 coding sequences. There were 3,814 protein-coding genes and 45 pseudogenes. The number of RNA genes was 58, including 6 rRNAs (2 5S, 2 16S, and 2 23S), 48 tRNAs, and 4 noncoding RNAs.

### Accession number(s).

This complete genome sequence has been deposited in GenBank under the accession number CP026747. The version described in this paper is the ﬁrst version.
